# Correction: GLYATL1 is associated with metabolic and epigenetic changes and with endocrine resistance in luminal breast cancer

**DOI:** 10.1186/s13148-026-02203-z

**Published:** 2026-07-10

**Authors:** Janina Müller, Emre Sofyali, Luisa Schwarzmüller, Yael Aylon, Eviatar Weizman, Lisa Schlicker, Katherine Kelly, Simone Borgoni, Simin Oz, Cole Stocker, Sara Burmester, Angelika Wörner, Sabine Karolus, Birgitta E. Michels, Daniela Heiss, Rainer Will, Veronica Rodrigues de Melo Costa, Pavlo Lutsik, Dieter Weichenhan, Ilse Hofmann, Nishanth Belugali Nataraj, Yosef Yarden, Luca Magnani, Christoph Plass, Almut Schulze, Cindy Körner, Moshe Oren, Stefan Wiemann

**Affiliations:** 1 Division of Molecular Genome Analysis, German Cancer Research Center (DKFZ), Im Neuenheimer Feld 580, 69120 Heidelberg, Germany; 2 Faculty of Biosciences, University of Heidelberg, Im Neuenheimer Feld 234, 69120 Heidelberg, Germany; 3 Department of Molecular Cell Biology, Weizmann Institute of Science, 76100 Rehovot, Israel; 4 G-INCPM, Weizmann Institute of Science, 76100 Rehovot, Israel; 5 Division of Tumor Metabolism and Microenvironment, German Cancer Research Center (DKFZ), Im Neuenheimer Feld 581, 69120 Heidelberg, Germany; 6 Division of Cancer Epigenomics, German Cancer Research Center (DKFZ), Im Neuenheimer Feld 280, 69120 Heidelberg, Germany; 7 Cellular Tools Core Facility, German Cancer Research Center (DKFZ), Im Neuenheimer Feld 580, 69120 Heidelberg, Germany; 8 Antibody Core Facility, German Cancer Research Center (DKFZ), Im Neuenheimer Feld 280, 69120 Heidelberg, Germany; 9 Department of Immunology and Regenerative Biology, Weizmann Institute of Science, 76100 Rehovot, Israel; 10 Present Address: Bugworks Research Inc., Center for Cellular and Molecular Platforms, National Center for Biological Sciences, TIFR GKVK Campus, Bellary Road, Bangalore, 560 065 India; 11 Department of Surgery and Cancer, Faculty of Medicine, Imperial College London, South Kensington Campus, London, SW7 2AZ UK; 12 Present Address: Breast Epigenetic Plasticity and Evolution Group, The Institute of Cancer Research, 123 Old Brompton Road, London, SW7 3RP UK

**Correction to: Clin Epigenet (2026) 18:76** 10.1186/s13148-026-02133-w

In article [1], The corresponding author inadvertently omitted the declaration of the Competing Interest in the original submission. This has now been corrected, and the statement has been added as indicated below.

The new Competing interest:

 “Stefan Wiemann is an Associate Editor of Clinical Epigenetics. To ensure an independent and unbiased review process, Stefan Wiemann had no involvement in the peer review, editorial assessment, or decision-making for this manuscript. The remaining authors declare no competing interests.”

The original article has been corrected.
