# Supplementary figures and images for: GLYATL1 is associated with metabolic and epigenetic changes and with endocrine resistance in luminal breast cancer
Source: Clin Epigenetics. 2026 Apr 29;18:76. doi: 10.1186/s13148-026-02133-w (PMC13126711; doi:10.1186/s13148-026-02133-w)

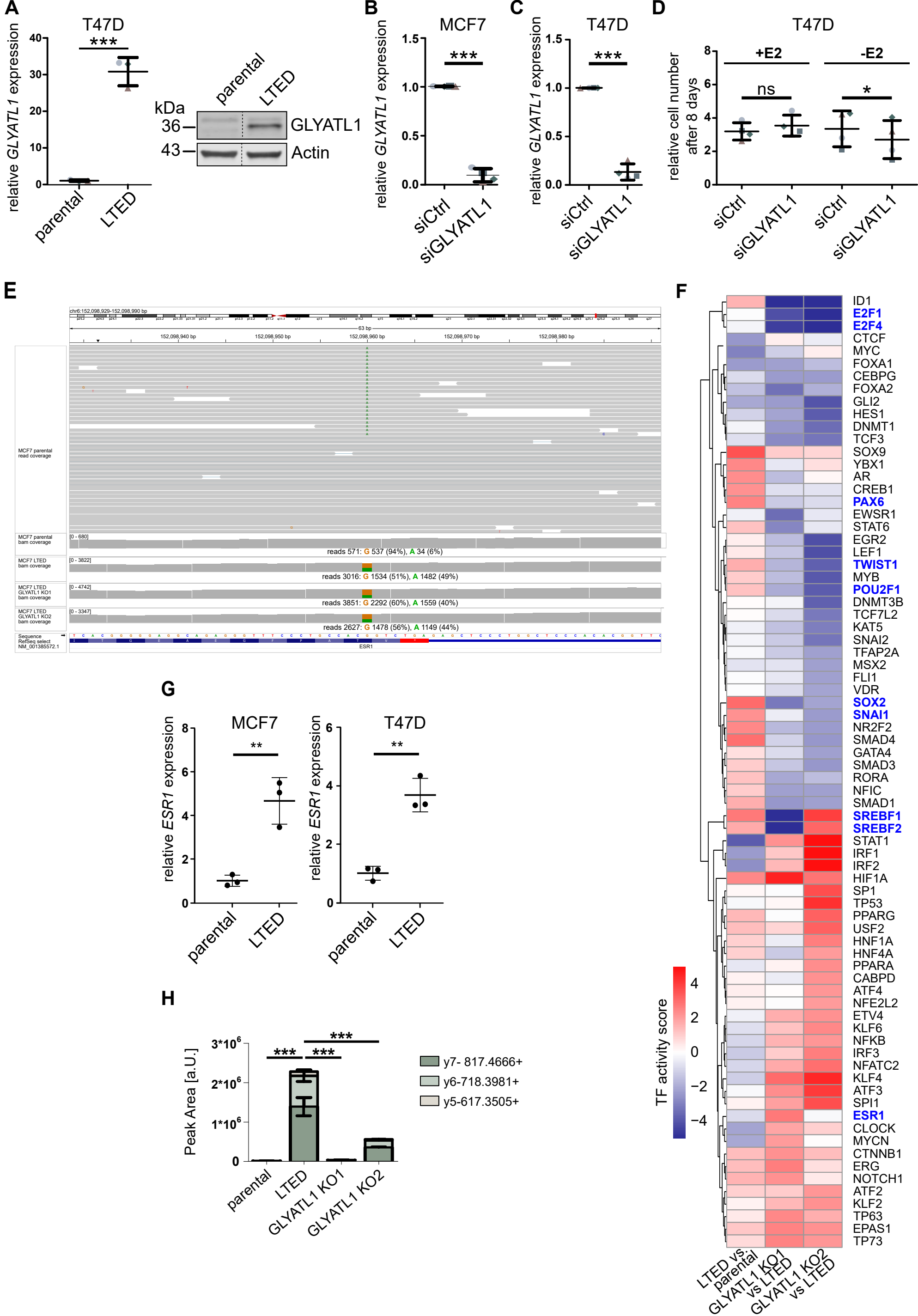

Supplement: Supplementary file 1 — Supplementary Material 1. Supplementary Figure 1: GLYATL1 is upregulated in long time estrogen deprived (LTED) T47D cell line, and GLYALT1 expression correlates with patient survival as well as affects transcription factor activities. (A) Relative GLYATL1 mRNA expression in T47D parental and long-term estrogen-deprived (LTED) cell lines analyzed by RT-qPCR (left). mRNA expression was normalized to ACTB and PUM1 expression and relative changes to the parental cells were calculated. Data are represented as the mean ± SEM (n=3). Statistical significance was assessed using unpaired Student's t-test, *** indicates p<0.001. GLYATL1 protein levels in T47D parental and LTED cells was analyzed by Western Blot (right). β-actin was used as a loading control. Uncropped images of blots are presented in the Supplementary File. (B) GLYATL1 was knocked down via RNA interference in MCF7 LTED cells and knockdown verified via RT-qPCR, compared to MCF7 LTED cells transfected with a non-targeting control siRNA (siCtrl). Data are represented as the mean ± SEM (n=3). Statistical significance was assessed using unpaired Student's t-test, *** indicates p<0.001. (C) GLYATL1 was knocked down via RNA interference in T47D LTED cells and knockdown verified via RT-qPCR, compared to T47D LTED cells transfected with a non-targeting control siRNA (siCtrl). Data are represented as the mean ± SEM (n=3). Statistical significance was assessed using unpaired Student's t-test, *** indicates p<0.001. (D) Equal numbers of T47D LTED control and LTED GLYATL1 knockdown cells were cultivated in media with (+E2) and without (-E2) estrogen for 8 days and cell numbers were then quantified via microscopy-based nuclear count and normalized to the respective seeding control. Statistical significance was assessed using paired Student's t-test. ns indicates non-significant p-value, * indicates p<0.05. (E) Read-coverage from RNA-sequencing in a window covering 63bp in the terminal exon of the ESR1 gene in MCF7 parental [file 13148_2026_2133_MOESM1_ESM.pdf]

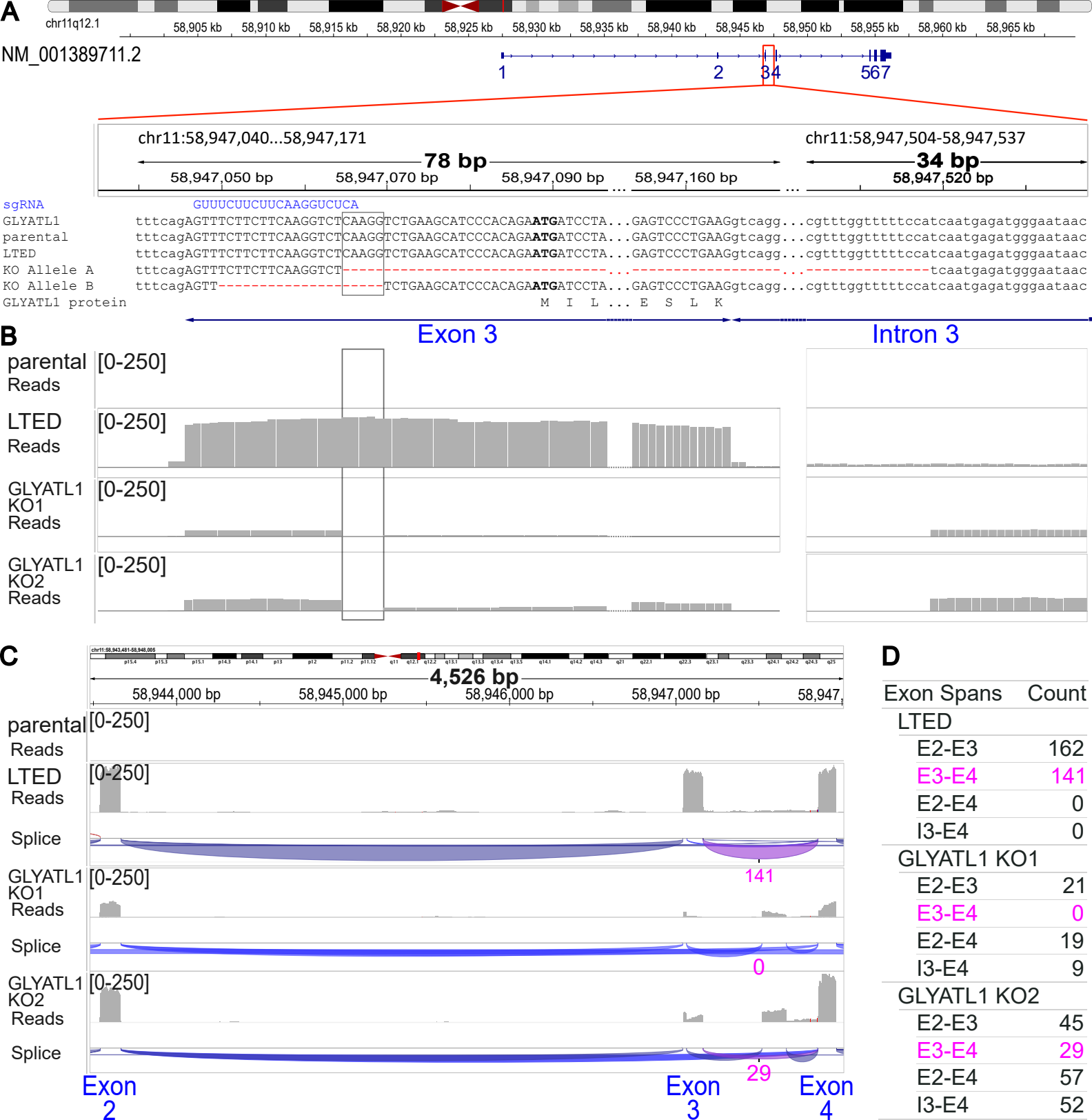

Supplement: Supplementary file 2 — Supplementary Material 2. Supplementary Figure 2: Genotyping and assessment of coding potential of LTED GLYATL1 knockout clones. (A) Gene structure (hg38) of GLYATL1 (Reference transcript NM_001389711.2) and sequences in the stretch (red open box) covering the 5’-end of exon 3 up to sequence in intron 3 obtained by genotyping (Sanger sequencing) of purified PCR-products from parental, LTED, and GLYATL1 knockout clones KO1/KO2. Note: PCR products from two knockout alleles (Allele A, B) were sequenced individually, and sequences were identical in GLYATL1 knockout clones KO1 and KO2. Exonic sequence is indicated in upper case letters of nucleic acid sequence while intronic sequences are in lower case. Sequences deleted in respective knockout alleles within exon 3 and around the distal breakpoint within intron 3 are indicated by red dashes. Intermittent sequences not shown are indicated by dots (…). A five bp overlap of deleted sequence in alleles A and B is indicated by a black open box. The sequence and position of the sgRNA binding site is indicated in blue. The translation start of the GLYATL1 open reading frame is indicated and the encoded amino acid sequence is shown below the nucleotide sequences. (B) Read coverage from GLYATL1 RNA-sequencing data is shown for parental, LTED, and GLYATL1 knockout clones KO1 and KO2. No sequence reads were detected in the parental condition. A five bp sequence without read-coverage in GLYATL1 knockout clones KO1 and KO2 is indicated by a black open box. Intermittent regions not shown are indicated by dots (…). Graphics adapted from Integrative Genomics Viewer (IGV) [90]. (C) Inferred splice junction patterns supported by reads from RNA-sequencing of MCF7 parental, LTED, and LTED GLYATL1 knockout clones KO1 and KO2. Read counts supporting splicing from exon 3 to exon 4 are indicated in pink for LTED, GLATLY1 KO1 and KO2 clones. Graphics adapted from IGV [90]. (D) Read coverage and splice junctions in RNA-sequencing data obtained fr [file 13148_2026_2133_MOESM2_ESM.pdf]

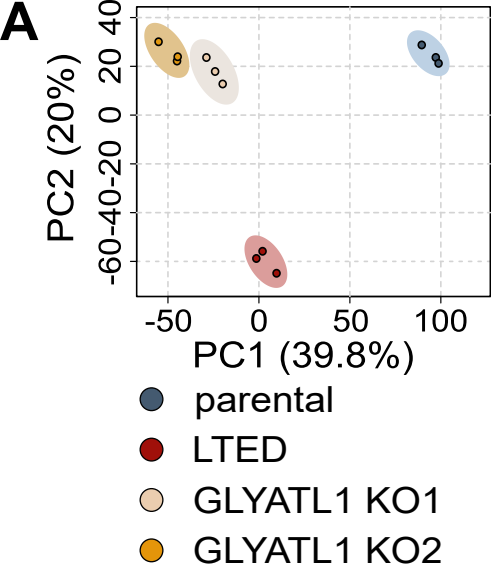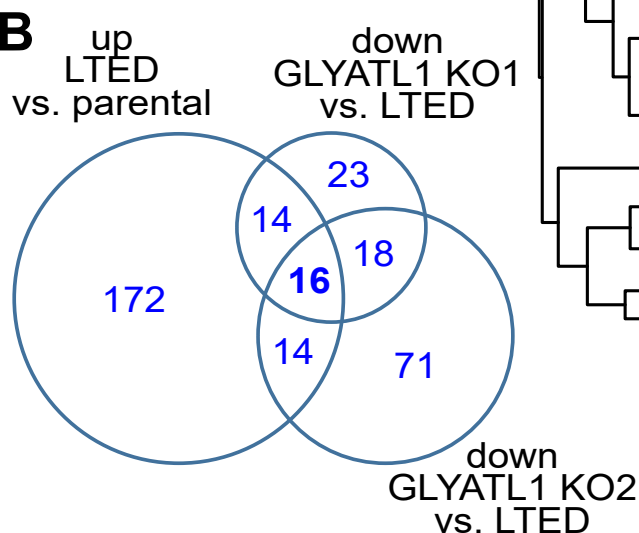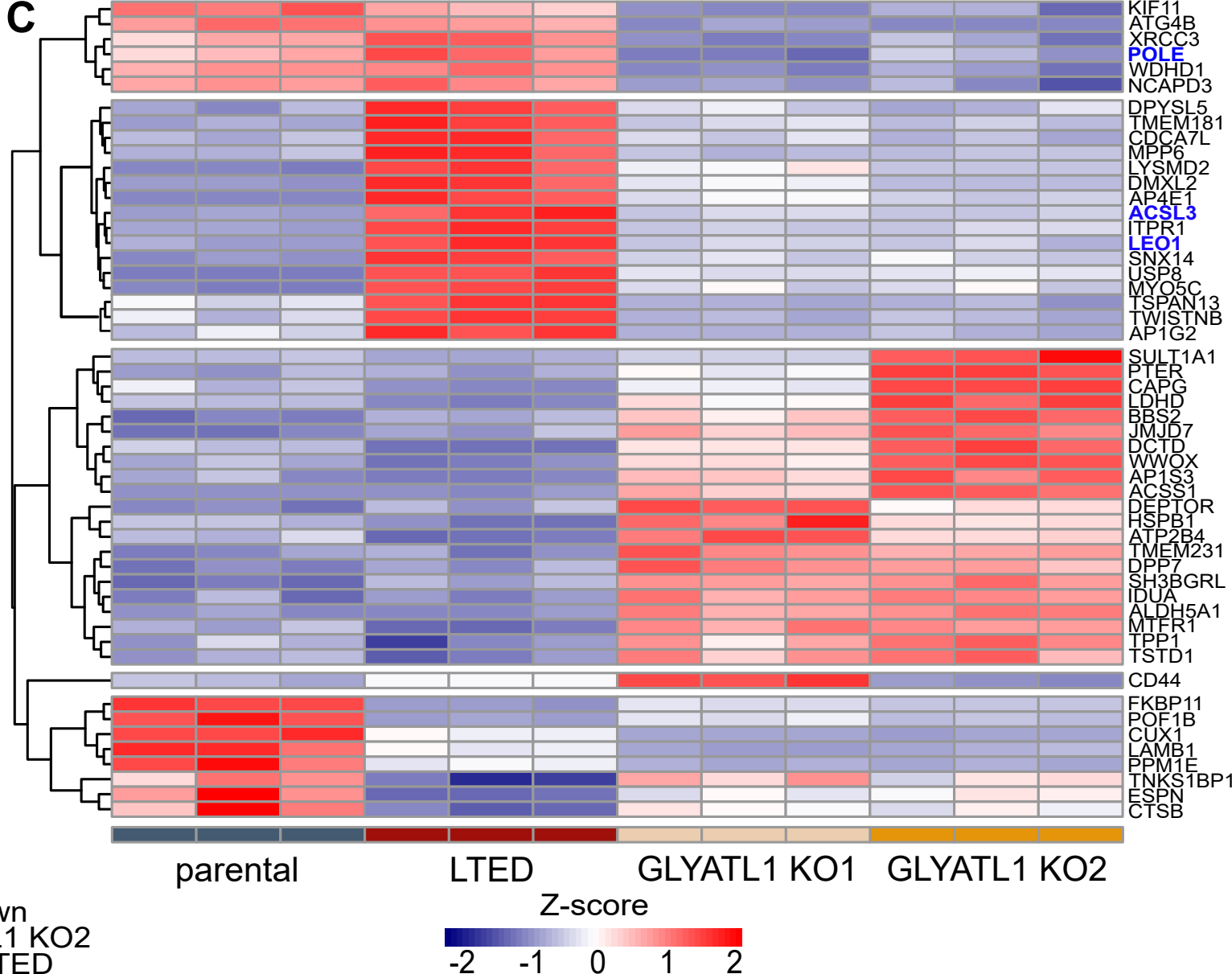

Supplement: Supplementary file 3 — Supplementary Material 3. Supplementary Figure 3: Proteomic changes associated with estrogen deprivation and with GLYATL1 protein expression. (A) Total proteins in MCF7 parental, long term estrogen deprived (LTED), and LTED GLYATL1 KO1 and KO2 cell lines were analyzed by mass spectrometry and protein intensities were used for principal component analysis. (B) VENN diagram depicting numbers of differentially expressed proteins, filtered for proteins significantly upregulated in MCF7 LTED vs. parental cells, and proteins significantly downregulated in MCF7 LTED GLYATL1 knockout clones KO1 or KO2 vs. LTED cells. Note: GLYATL1 is not listed as this protein was detected neither in parental nor in LTED GLYATL1 KO1 cells. Statistical significance was determined by Student’s unpaired t-test and p-values were adjusted using the Benjamini-Hochberg method. Thresholds: log2FC >1 (LTED vs. parental) and <-1 (KO vs. LTED); significance: adjusted p-value < 0.05. (C) Hierarchically clustered heatmap showing z-scaled intensities of proteins with significant changes (adjusted p-values < 0.05) in LTED GLYATL1 KO1 and KO2 cell lines compared to parental or long-term estrogen deprived (LTED) MCF7 cell lines, with an absolute log2 fold-change of at least 1 in any comparison. Biological replicates (n=3) are displayed separately. Statistical significance was determined by Student’s unpaired t-test and p-values were adjusted using the Benjamini-Hochberg method. [file 13148_2026_2133_MOESM3_ESM.pdf]

A

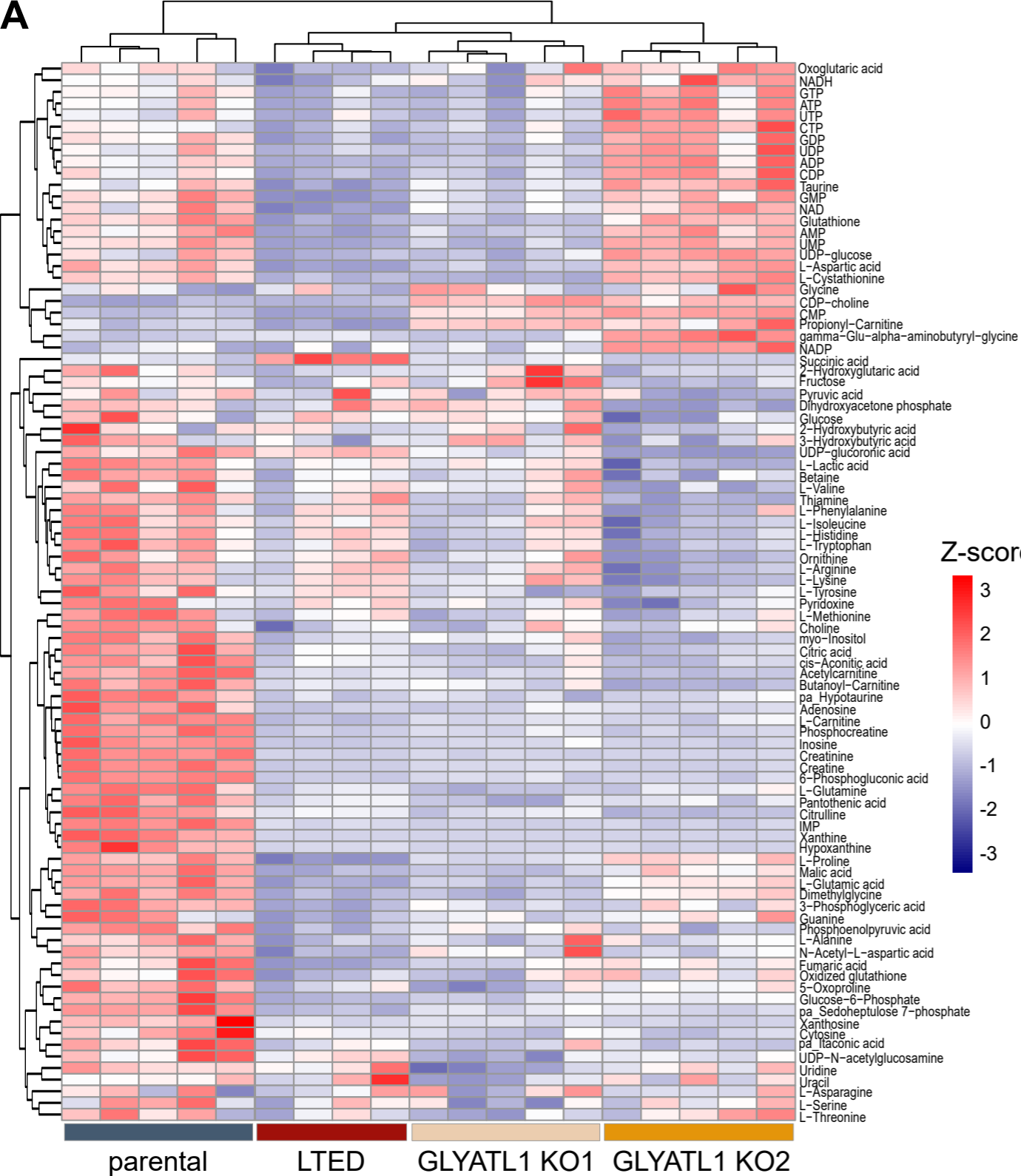

B

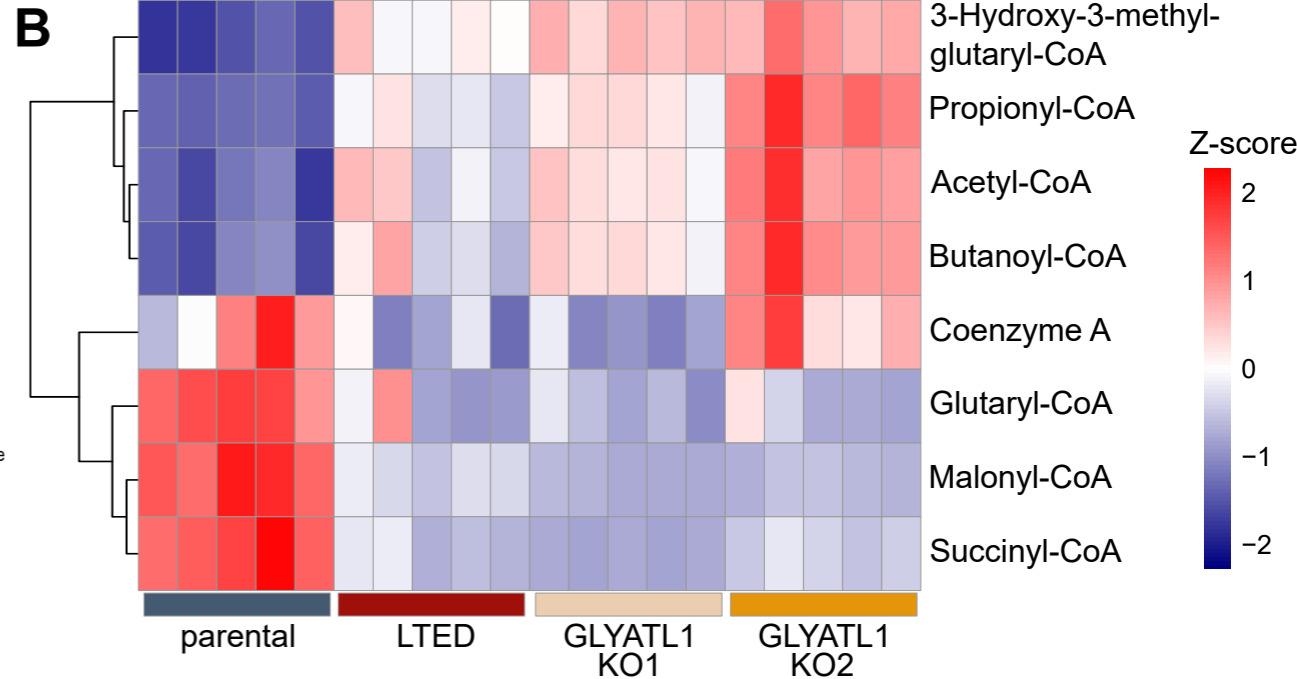

C

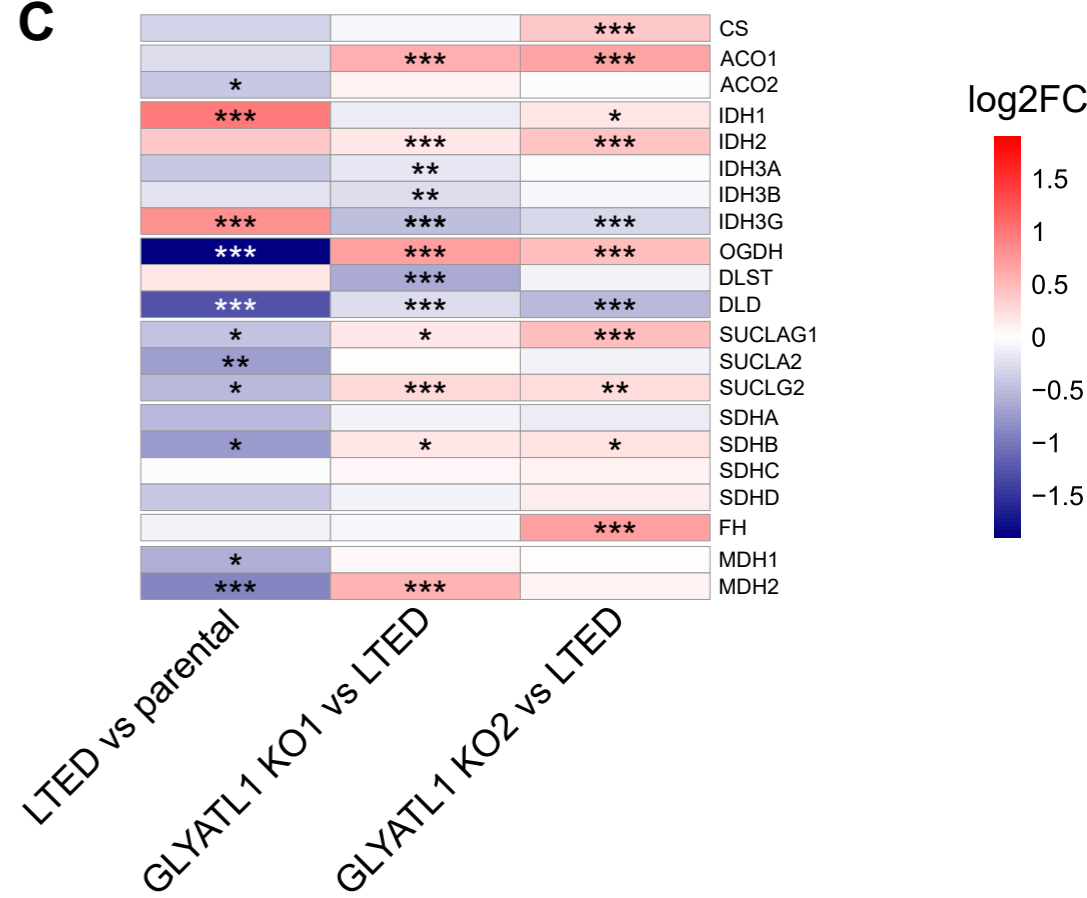

Supplement: Supplementary file 4 — Supplementary Material 4. Supplementary Figure 4: Metabolomic analysis of MCF7 parental and long-term estrogen deprived (LTED) cells, and in LTED GLYATL1 knockout clones KO1 and KO2. (A) Hierarchical clustering showing z-scaled intensities, normalized by their respective internal standard levels, of soluble metabolites that were measured by mass spectrometry in MCF7 parental, LTED, and the LTED GLYATL1 knockout clones KO1 and KO2. Biological replicates (n≥4) are displayed individually. (B) Heatmap showing z-scaled values of acyl-CoA species measured via LC-MS in MCF7 parental and LTED cells, and two GLYATL1 knockout clones KO1 and KO2 (n=5). (C) Differential gene expression (RNA-sequencing) of indicated genes in the TCA-cycle as determined by DESeq2 analysis, sorted by their respective position in that cycle. Data extracted from Supplementary Tables 1 and 3. * indicates Benjamini-Hochberg adjusted p<0.05, ** indicates p<0.01, and *** indicates p<0.001. [file 13148_2026_2133_MOESM4_ESM.pdf]

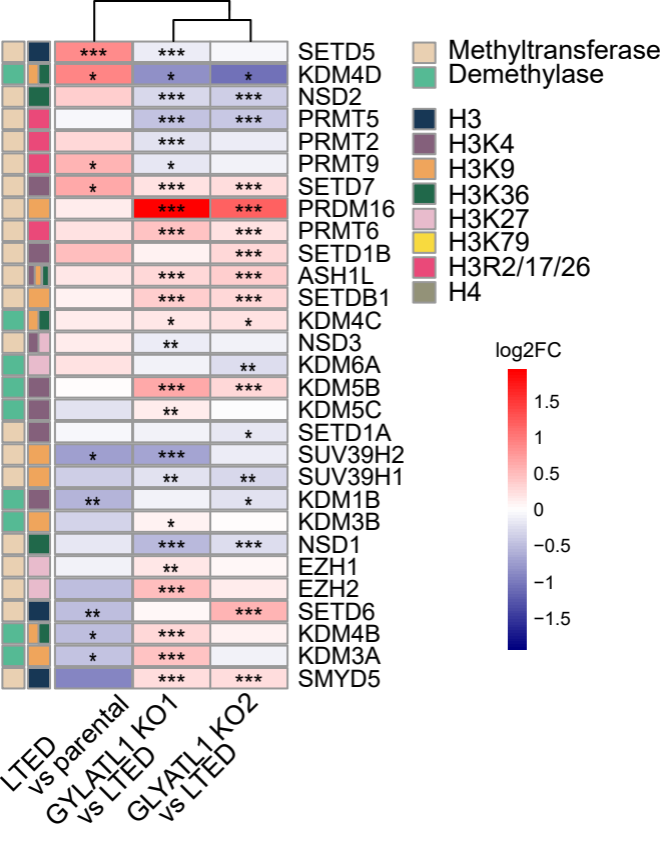

Supplement: Supplementary file 5 — Supplementary Material 5. Supplementary Figure 5: Differentially expressed methyltransferases and demethylases in MCF7 long-term estrogen deprived (LTED) vs. parental cells, and in LTED GLYATL1 knockout clones KO1 and KO2 vs. LTED cells. Heatmap displays log2 fold-changes (log2FC) in mRNA levels of genes encoding histone modifiers affecting methylation status as measured by RNA sequencing of MCF7 parental and long-term estrogen deprived (LTED) cells, and two LTED GLYATL1 knockout clones KO1 and KO2, followed by DESeq2 analysis. Respective comparisons are indicated below the heatmaps. Writers of histone marks are indicated in beige and erasers are indicated in green. Affected histone residues are indicated in colors for the respective epigenetic modifiers. * indicates Benjamini Hochberg adjusted p<0.05, ** indicates adjusted p<0.01, and *** indicates adjusted p<0.001. [file 13148_2026_2133_MOESM5_ESM.pdf]

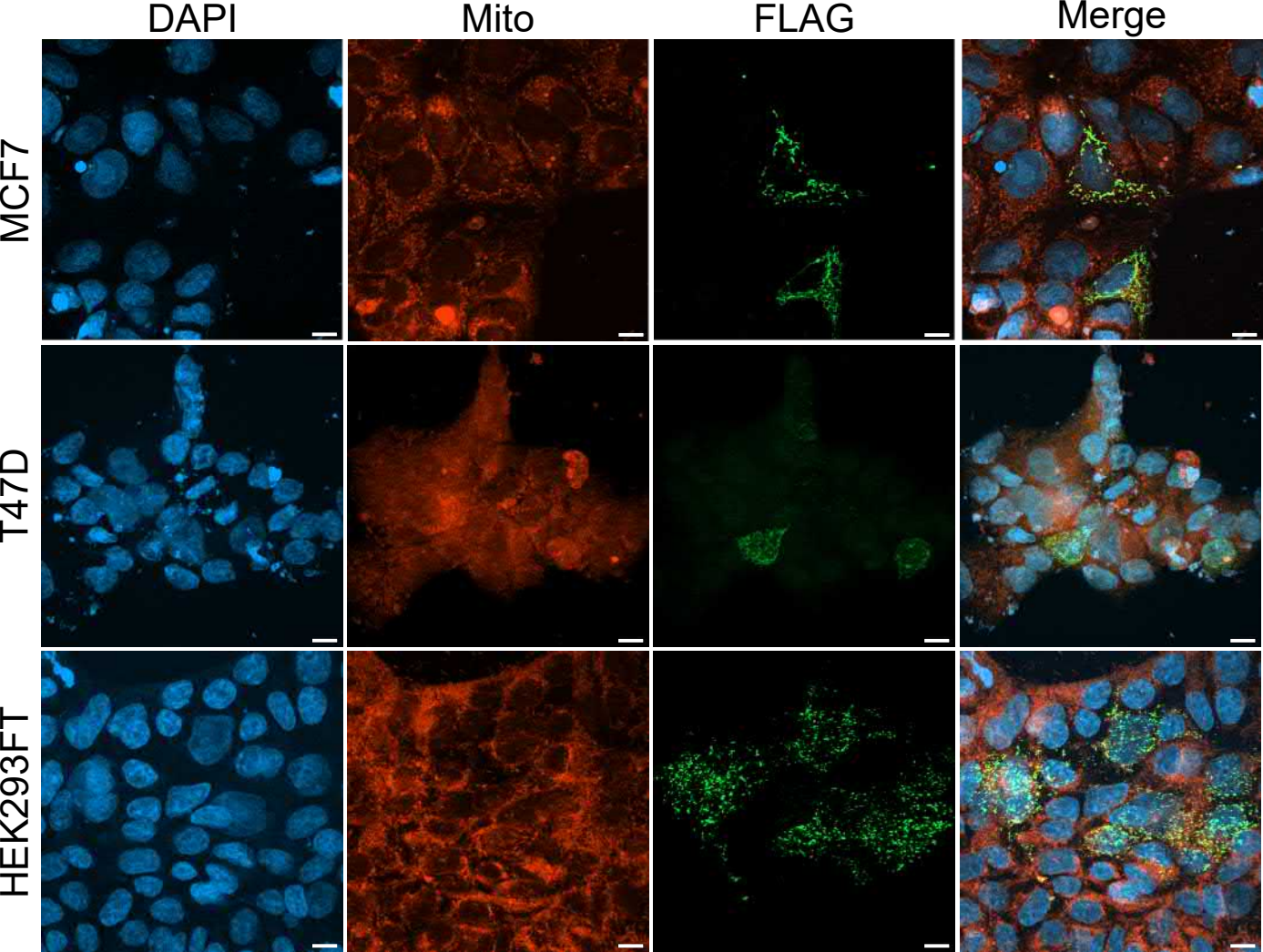

Supplement: Supplementary file 6 — Supplementary Material 6. Supplementary Figure 6: GLYATL1 protein localizes in the mitochondria in MCF7, T47D and HEK293FT cell lines. C-terminally FLAG-tagged GLYATL1 was recombinantly overexpressed in MCF7, T47D and HEK293FT cell lines by transient plasmid transfection. MCF7 and T47D cells were cultivated for 72 hours after transfection and HEK cells for 48 hours, and then incubated with abberior LIVE ORANGE dye to stain mitochondria (Mito). Cells were fixed and incubated with an anti-Flag antibody (FLAG) to detect the GLYATL1-FLAG fusion protein. Finally, nuclei were stained with DAPI and images taken using confocal immunofluorescence microscopy. Image analysis was performed using Zen Blue software and ImageJ (https://imagej.net/ij/). Representative images are shown. Scale bar = 10 µm. [file 13148_2026_2133_MOESM6_ESM.pdf]

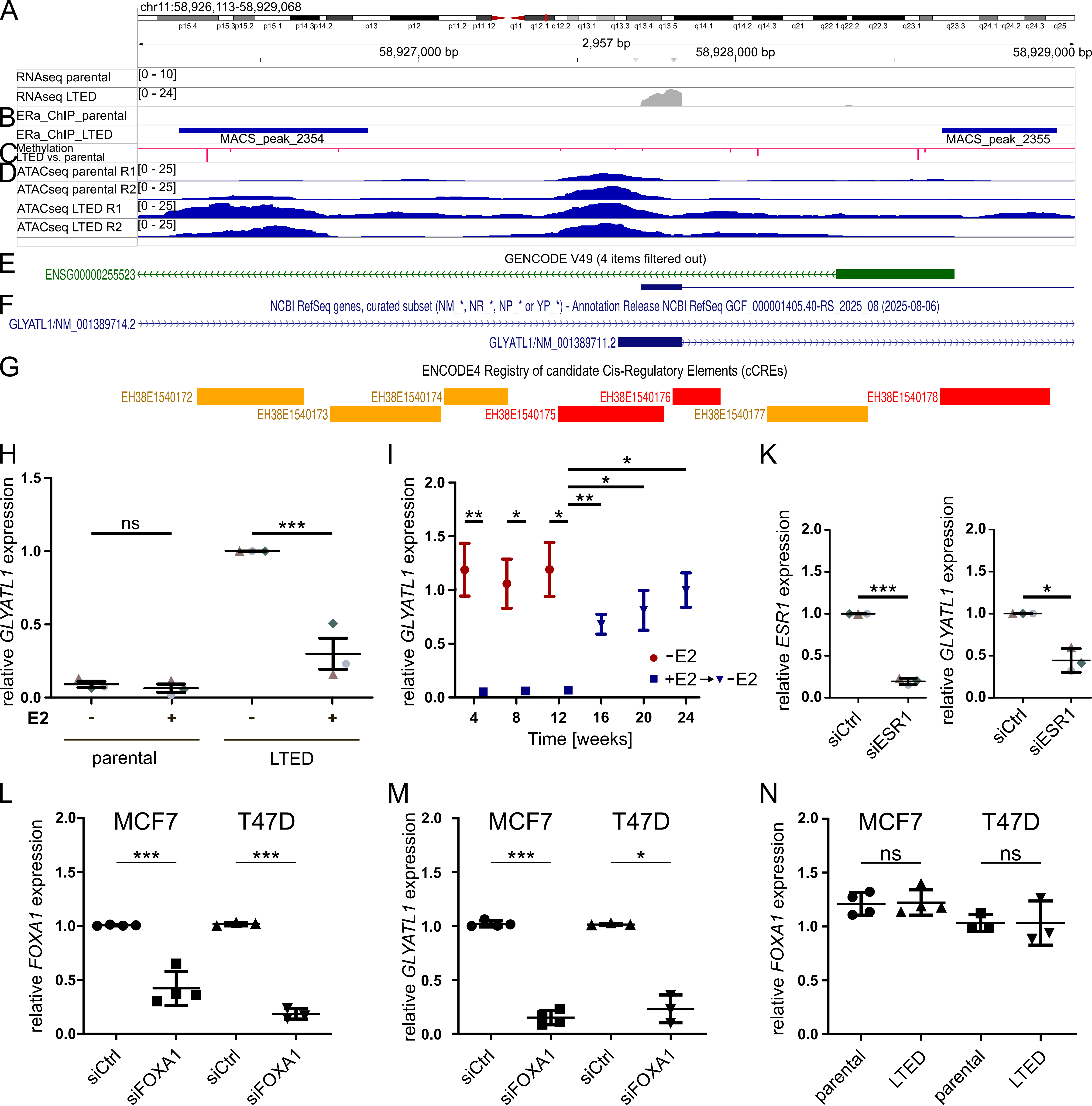

Supplement: Supplementary file 7 — Supplementary Material 7. Supplementary Figure 7: Regulation of GLYATL1 gene expression. Genomic mapping of regulatory sequence in the GLYATL1 gene locus (GRCh38/hg38) proximal and distal of the transcription start site. Shown is read coverage in RNA-seq data (A), data from ERα ChIP-seq (MACS_peaks from GSE60517 [20]) (B), methylation changes of LTED vs. parental condition (EPIC-array) (C), and ATAC-seq peaks acquired from MCF7 parental and long time estrogen-deprived (LTED) cells (D). Gencode (E) and NCBI RefSeq (F) tracks from the UCSC genome browser mapping at the genomic region. (G) ENCODE4 Registry of candidate Cis-Regulatory Elements (cCREs) with putative enhancers indicated in yellow and putative promoters indicated in red. In (C), red and blue peaks depict hypermethylated and hypomethylated CpG positions, respectively, in the LTED cells compared to parental MCF7. Panels E-G have been adapted from the UCSC genome browser [22]. (H) Parental T47D cells (left) and T47D LTED cells (right) were cultivated for 48 hours in media without (-) or with (+) supplementation of 10 nM 17-β-estradiol (E2). Then, RNA was extracted and GLYATL1 mRNA levels were assessed via RT-qPCR. Data from all conditions were normalized to LTED cells cultivated in estrogen-depleted media (n=3, each with 3 technical replicates). Statistical significance was assessed using one-way ANOVA with Bonferroni post-test. ** indicates p<0.01, ns: not significant. (I) T47D LTED cells were cultured in the presence (+E2, blue squares) or absence (-E2, red circles) of estrogen for 12 weeks. After these initial 12 weeks, cells were deprived of estrogen again and cultivation was continued for another 12 weeks (+E2 -> -E2, black triangles). mRNA levels were determined by RT-qPCR from cultures harvested at the indicated time points. Relative changes to LTED cultivated in estrogen-deprived media were calculated (n≥4, with 3 technical replicates each). Statistical significance was assessed using unpaired Student [file 13148_2026_2133_MOESM7_ESM.pdf]
