# Supplementary material for: GLYATL1 is associated with metabolic and epigenetic changes and with endocrine resistance in luminal breast cancer
Source: Clin Epigenetics. 2026 Apr 29;18:76. doi: 10.1186/s13148-026-02133-w (PMC13126711; doi:10.1186/s13148-026-02133-w)
Supplement: Supplementary file 8 — Supplementary Material 8. Supplementary File: uncropped Western blots, validation of GLYATL1 antibody and initial validation of MCF7 LTED GLYATL1 knockout clones. Lysates from indicated MCF7 and T47D cell lines were separated by SDS-PAGE and blotted onto a PVDF membranes. The membranes were blocked for unspecific binding of proteins and then incubated with a primary anti GLYATL1 antibodies in blocking buffer. The next day, membranes were washed three times. Then, the blots were incubated with a secondary antibody, which was conjugated with Alexa Fluor™ 680, and washed again. Proteins were visualized with a LI-COR Odyssey scanner using 700 and 800 nm channels. Then, the same blots were reprobed with an anti ß-Actin antibody over night. The next day, the blots were washed and then incubated with a secondary antibody conjugated with DyLight™ 800 4X PEG. Protein bands were again visualized with a LI-COR Odyssey scanner using 700 and 800 nm channels. Original scans and accompanying files are provided in a supplementary ZIP-archive. (A) Cropped images (MCF7 from Figure 1B and T47D from Supplementary Figure 1A) are shown in the top left. The respective uncropped Western blot images of the original gel with indication of molecular weight markers (PageRuler Protein Ladder) are shown on the right. Samples from several MCF7 and T47D cell lines and derivatives were separated on the same gel. Lanes 2 and 4 (MCF7) and 7 and 9 (T47D) were cropped to generate the final images. Other conditions that were tested (i.e., TAMR and LTED-TAMR for MCF7 [20], TAMR for T47D [21]) were not regarded in the current study. (B) Protein lysates of indicated cell lines were generated on the indicated dates and analyzed by SDS-PAGE and Western blot. The same marker was used as in panel A. (C) Protein lysates from GLYATL1 knockout clones generated from MCF7 TAMR and LTED cells were tested for expression of GLYATL1 protein. Lysates from MCF7 TAMR and LTED cells were used as positive control for GLYATL [file 13148_2026_2133_MOESM8_ESM.pdf]

A: Originals of cropped images

uncropped images

MCF7
Figure 1B

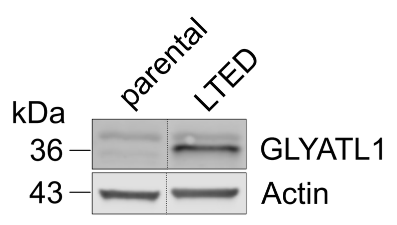

T47D
Supplementary
Figure 1A

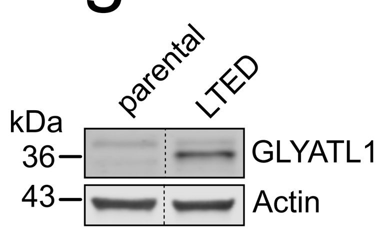

PageRuler™ Prestained Protein Ladder 10-180 kDa
Order number: 26616
Thermo Fisher Scientific, Waltham, MA, USA

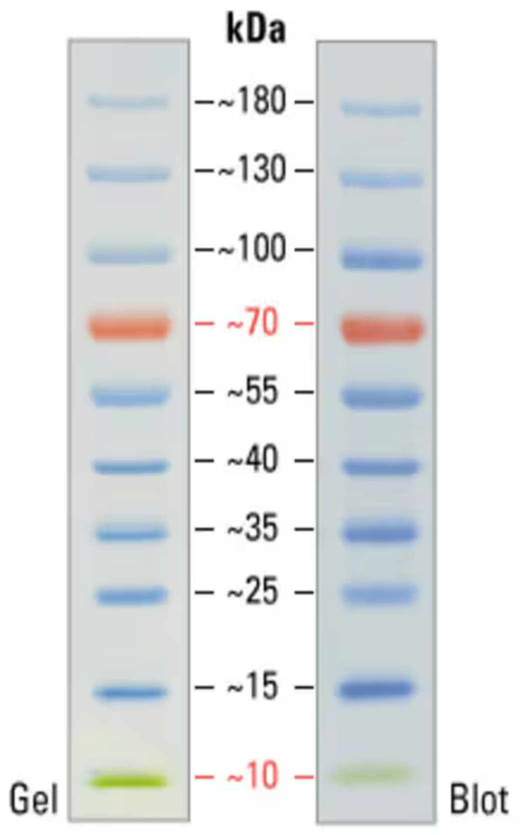

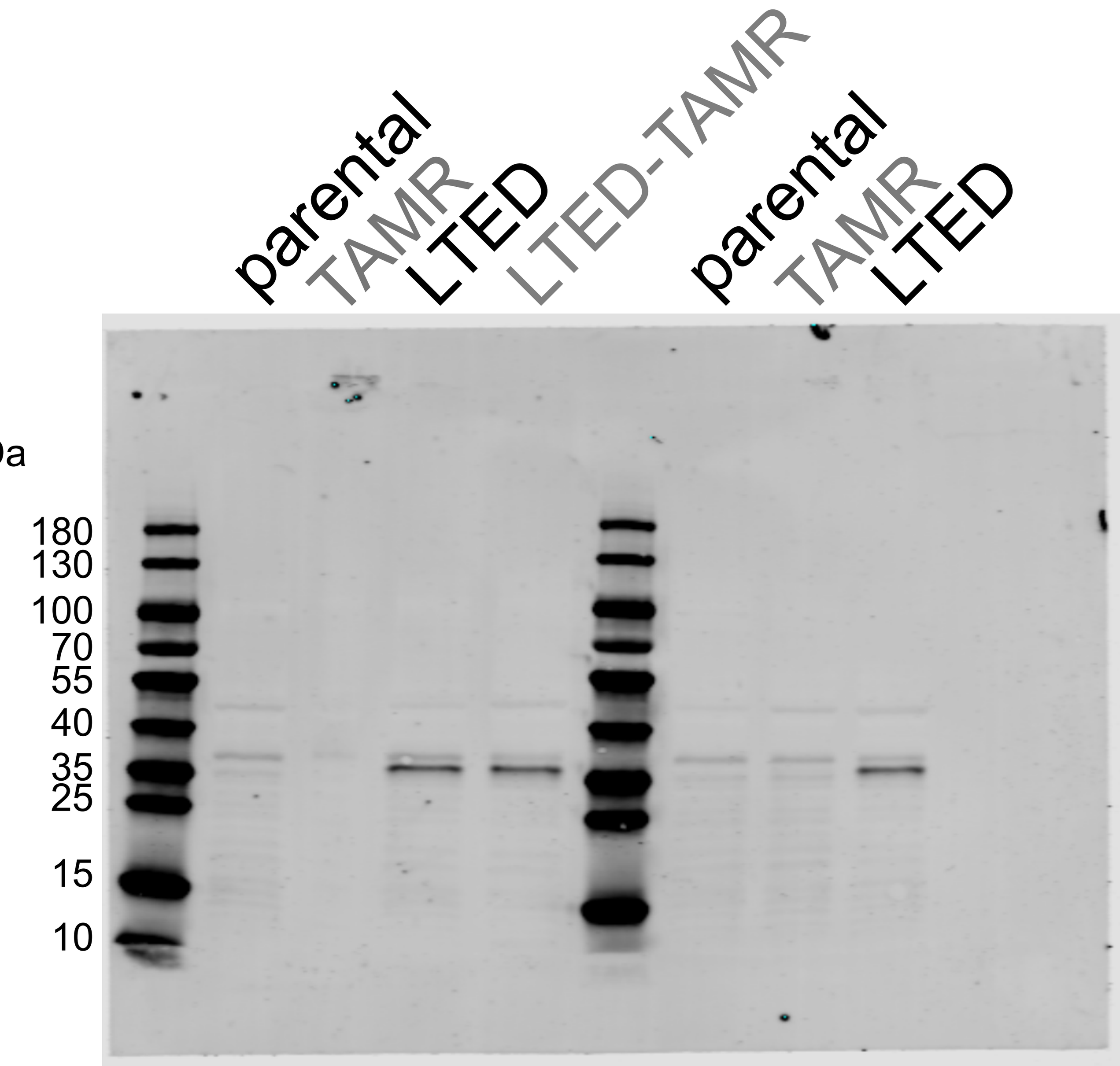

anti-GLYATL1
Dilution: 1:1000
Order number: HPA039501
Vendor: Sigma-Aldrich, Taufkirchen, Germany

Western blot was scanned on 15.06.2021

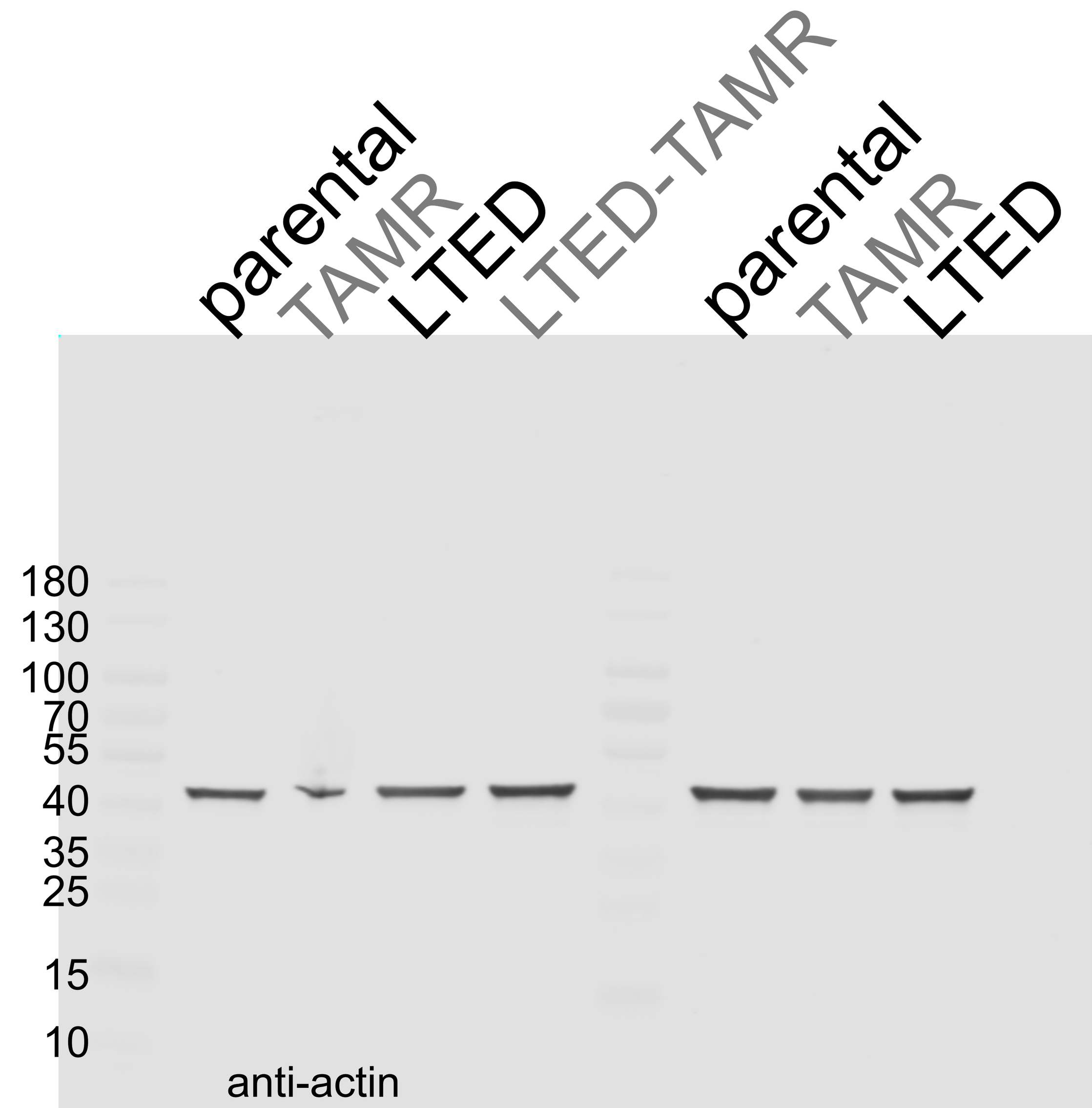

anti-actin
Dilution: 1:10,000
Order number: 0869100-CF
Vendor: MP Biologicals, Irvine, CA, USA

Notes:
TAMR: Cell lines and nomenclature (TAMR, LTED, LTED-TAMR) from Nguyen et al., PMID:26610607
T47D: Cell lines and nomenclature (TAMR, LTED) from Borgoni et al., PMID:33050633
TAMR and LTED-TAMR cell lines (in gray) not used in this study

B: Replication of Western blot. Protein lysates were generated on different dates

Loading scheme

1 • PageRuler Marker
2 • MCF7 parental 18.06.2021
3 • MCF7 TAMR 25.06.2021
4 • MCF7 LTED 25.06.2021
5 • MCF7 LTEDTAMR 25.06.2021
6 • MCF7 parental 16.06.2021
7 • MCF7 TAMR 15.06.2021
8 • MCF7 LTED 15.06.2021
9 • MCF7 LTEDTAMR 15.06.2021
10 • MCF7 LTED siCtrl
11 • MCF7 LTED siGLYATL1

dates indicate the day when respective protein lysates were generated

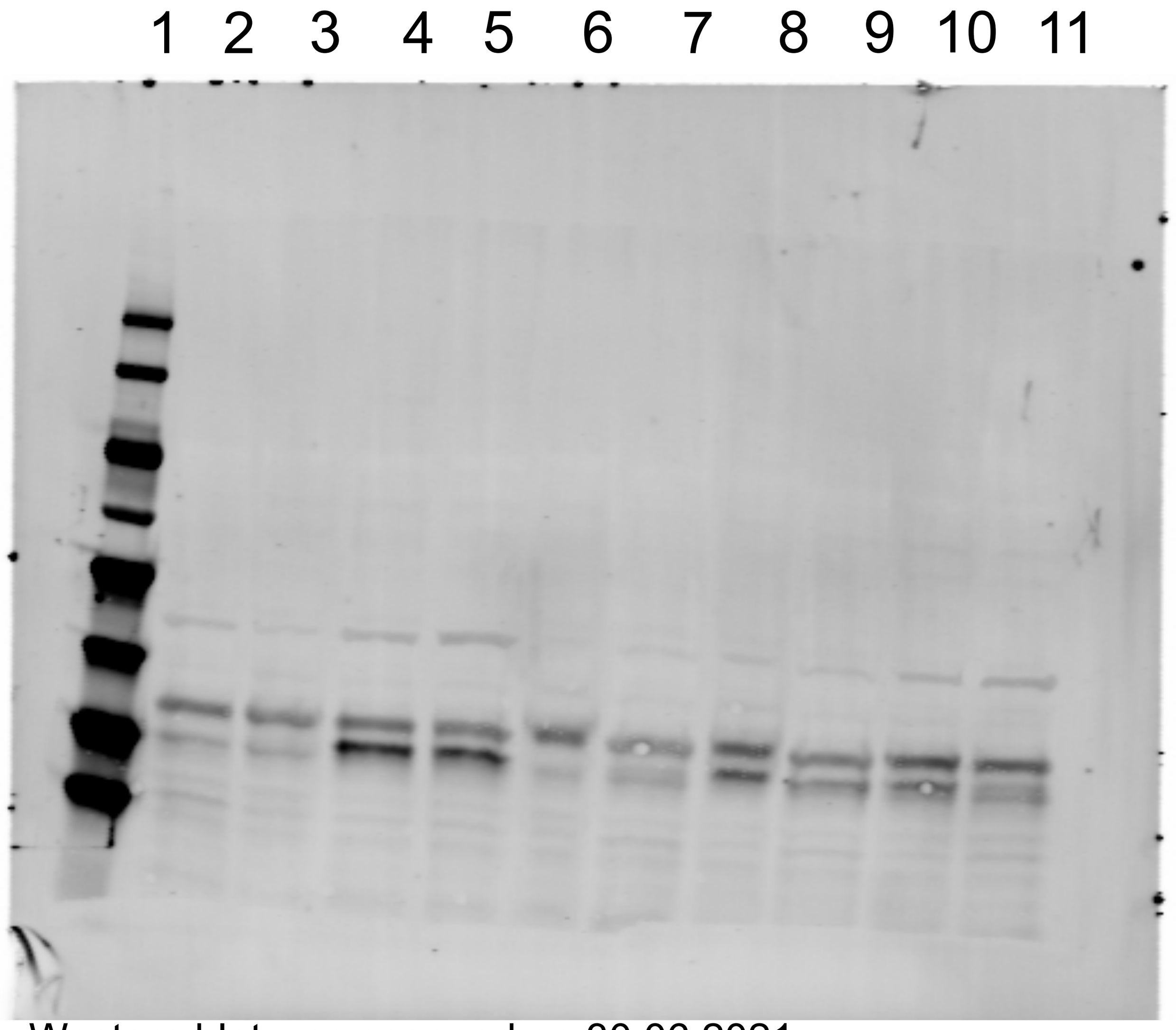

Western blot was scanned on 30.06.2021

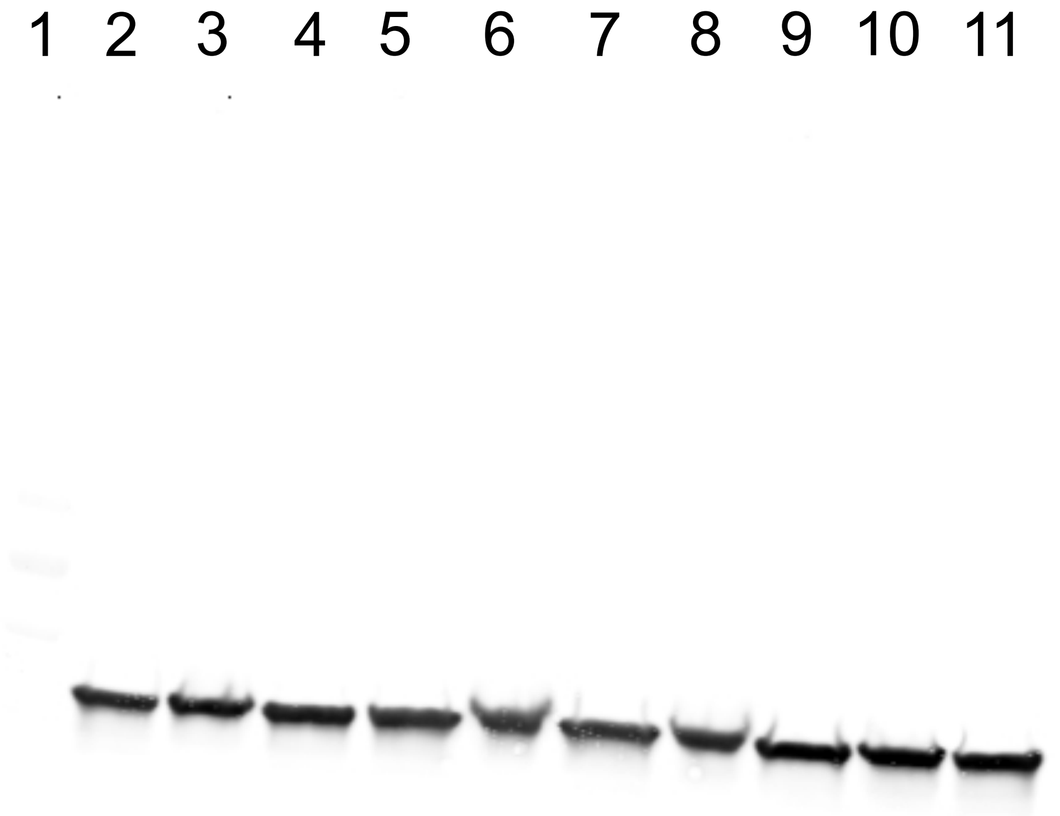

Notes:
TAMR: Cell lines and nomenclature (TAMR, LTED, LTED-TAMR) from Nguyen et al., PMID:26610607
TAMR and LTED-TAMR cell lines not used in this study
Dates indicate the day when respective protein lysates were generated

anti-GLYATL1
Dilution: 1:1000
Order number: HPA039501
Vendor: Sigma-Aldrich, Taufkirchen, Germany

anti-actin
Dilution: 1:10,000
Order number: 0869100-CF
Vendor: MP Biologicals, Irvine, CA, USA

C: Validation of GLYATL1 antibody and of *GLYATL1* knock-out clones

Loading scheme

1 • PageRuler Marker
2 • MCF7 TAMR 25.06.2021
3 • MCF7 TAMR KO1 P1 D4
4 • MCF7 TAMR KO2 P1 G3
5 • MCF7 TAMR KO3 P3 D1
6 • MCF7 TAMR KO4 P3 E6
7 • MCF7 LTED 25.06.2021
8 • MCF7 LTED KO1 P1 G4
9 • MCF7 LTED KO3 P1 G9
10 • MCF7 LTED KO2 P3 G4

final nomenclature - original nomenclature
LTED GLYATL1 KO1 - plate 1 position G4
LTED GLYATL1 KO2 - plate 3 position G4

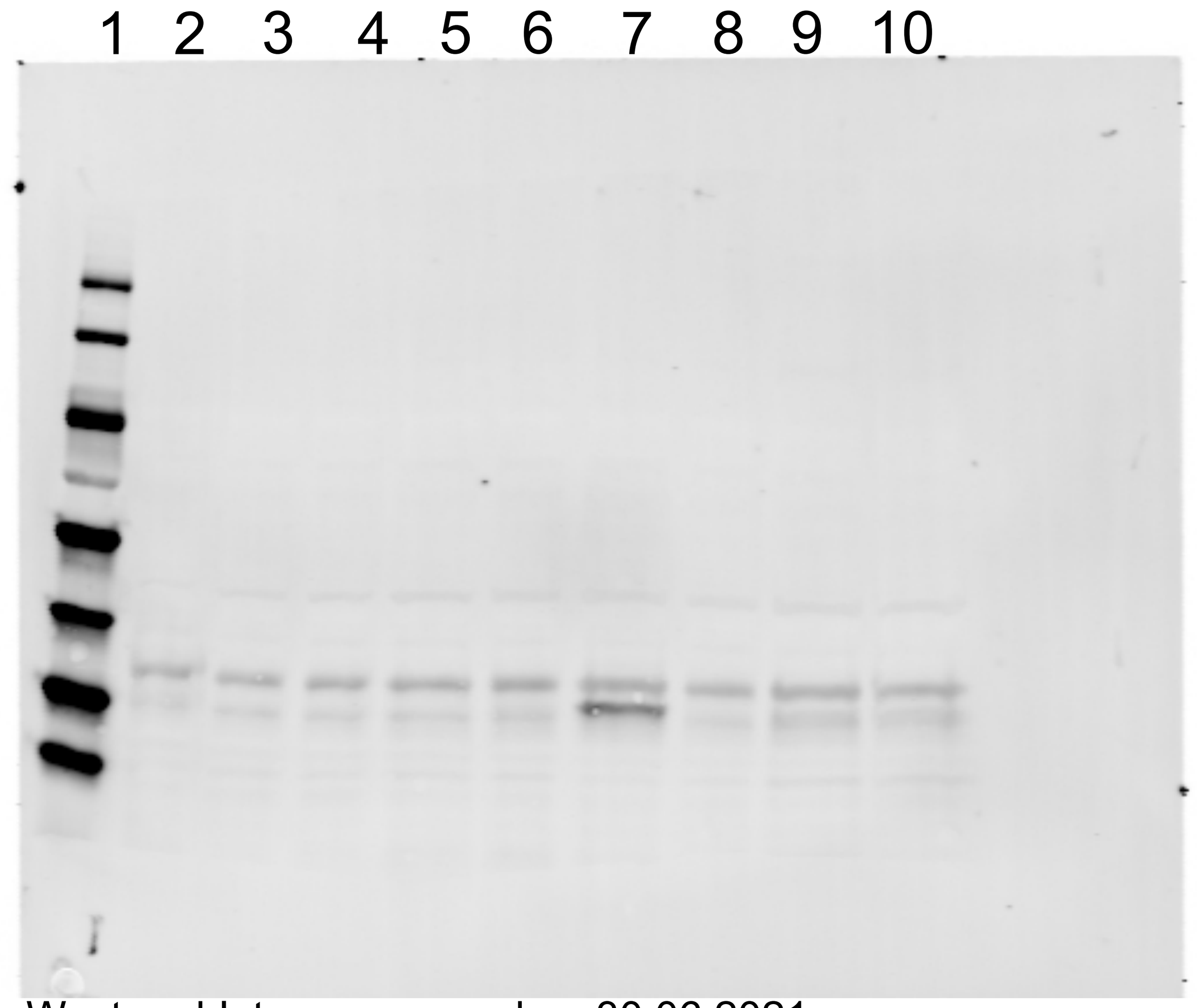

Western blot was scanned on 30.06.2021

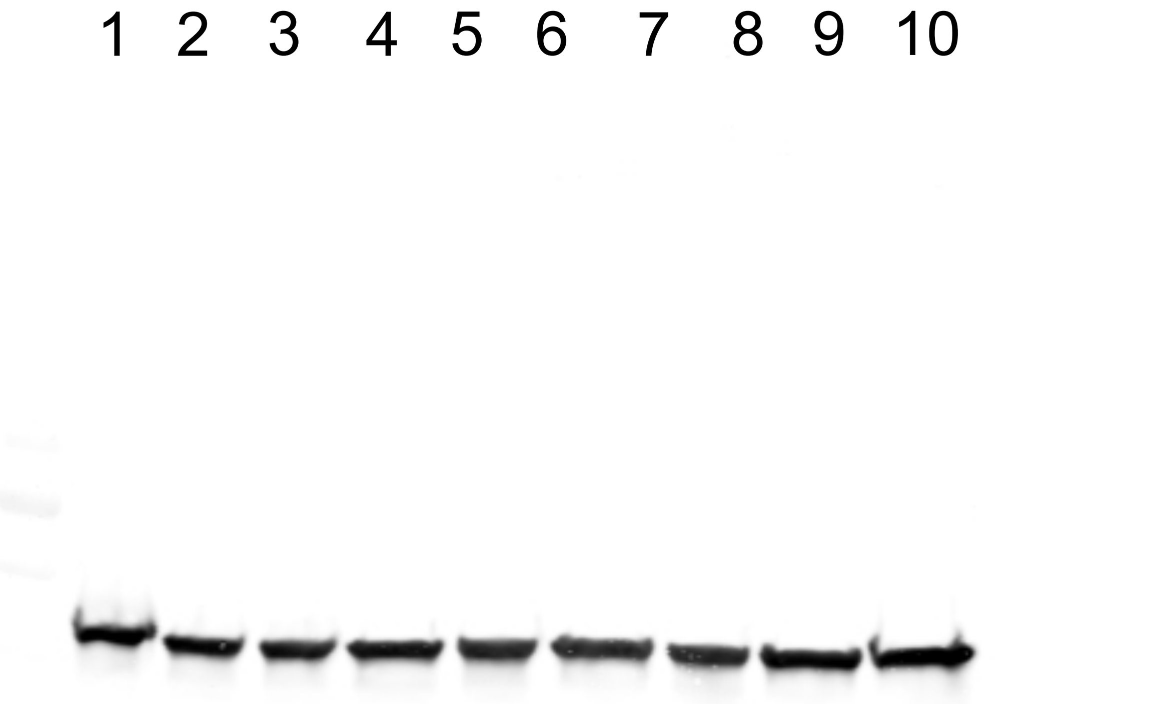

Notes:
TAMR: Cell lines and nomenclature (TAMR, LTED) from Nguyen et al., PMID:26610607
TAMR *GLYATL1* knockout cell lines were not used in this study
GLYATL1 knockout clone KO3 P1 G9 was not used in this study
Dates indicate the day when respective protein lysates were generated

anti-GLYATL1
Dilution: 1:1000
Order number: HPA039501
Vendor: Sigma-Aldrich, Taufkirchen, Germany

anti-actin
Dilution: 1:10,000
Order number: 0869100-CF
Vendor: MP Biologicals, Irvine, CA, USA
